# Unqualified Practice, the Medical Profession and the Public

**Published:** 1908-02-08

**Authors:** 


### The Hospital

A Journal of

The Medical Sciences and Hospital Administration.

New Series. No. 47, Vol. II. [No. 1119, Vol. XLIII.] SATURDAY, FEBRUARY 8, 1908.

### Unqualified Practice, the Medical Profession and the Public.

It is no new discovery that the public view of unqualified practice is very different from that which is held generally on the same subject by members of the medical profession. The difference is doubtless due in part to the fact that the shield is regarded purely or mainly from opposite sides. The layman hears only of wonderful cures and striking successes, whilst of the risks which are run in the attainment of these he is wholly ignorant. Whether the diagnosis of the condition for which relief has been obtained was, or was not, accurate, does not concern him. He judges solely by results as announced by persons admittedly innocent of the methods of scientific medicine and surgery, and incapable of judging accurately of complex pathological states. Further, the results which reach the public ear are mainly, or even entirely, those which are, or appear to be, of a gratifying character. The patient who is not relieved by the unqualified practitioner, or who is injured by the methods adopted in such practice, is inclined to keep his grievance to himself, or at least to abstain from anything in the shape of a public announcement, as he realises that his conduct may excite the comment of some caustic tongue. Add to these considerations the fact that the majority of mankind have a sort of sub-conscious belief that in the treatment of disease there is an element of mystery, to the secret of which only the initiated can attain, and it is not difficult to understand the wide extent and favour which unqualified practice commands in all ranks of the community. The layman judges the winning of tricks to be a proof of good play; he hears of successes, but not of risks and failures; and the claim of special and peculiar abilities and powers accords with his prepossession of the mysterious character which, as he thinks, is necessarily attached to curative methods.

The other side of the shield, on the contrary, is the one which presents itself to members of the medical profession. The practitioner is aware from his training and experience of the risks which are involved by many of the methods adopted by those who undertake the treatment of patients without adequate scientific and clinical training. He knows, also, at least in some measure, of the extent to which these methods fail, or even prove disastrous. And while allowing that good is done in individual instances, he is compelled to recognise there are always possibilities of mischief and danger even though numerous cases can be quoted in which these possibilities have not been realised. There is another element which contributes to the professional attitude towards unqualified practice. This is found in the existence in such practice of much pretence, humbug, and fraud. No one can deny that by deliberate and wilful deceit many persons in all ages have been fleeced and robbed by quacks, and even allowing that some unqualified practitioners are honestly convinced of the efficacy of the methods they profess and the medicines they offer, there are others who are nothing but parasites and plunderers of the public. For these and other reasons the medical profession has no option but to proclaim the risks and dangers which, in the very nature of the case, are attached to the diagnosis and treatment of disease by unqualified persons.

Endeavouring now to look at these two different points of view from a detached position, and listening to the case presented both by one side and the other, may it not be asked whether some element of exaggeration does not enter into each of the rival statements? Our readers will readily allow that this is true of the position occupied by many who proclaim the virtues and successes of the unqualified practitioner, and we quite agree that here exaggeration often reaches a high level, and even escapes beyond the bounds of veracity. But is the case for orthodox and common-sense medicine always presented with accuracy and precision? With all deference we will venture to answer this question in the negative. The medical practitioner, we have already suggested, sees for the most part one side of the shield, and there is much in that aspect, it must be admitted, calculated to stir the blood of honest and self-respecting men. Hence it is not altogether to be wondered at that it calls forth vigorous denunciation. The evil exists, and it is necessary in the public interest that they should be exposed. Yet even here it is well that attitude and expression should alike be distinctly judicial. By all means let the dangers and risks of unqualified practice be proclaimed, but in so doing let the colouring and perspective of the picture be strictly in accordance with fact. It is useless, and worse than useless, to denounce as dangerous and full of risk a method or policy which general experience appears largely to justify. This is illustrated in the extreme statements which are often made by those who condemn absolutely the use of alcohol. To such statements the great body of the public, arguing from direct experience, turns an inattentive ear. Exactly the same thing happens, and will continue to happen, to similar proclamations regarding other questions bearing on health and on the treatment of disease. There are methods of unqualified practice which are largely adopted with comparatively little risk, and to overstate the risk is to spoil the case against such methods. The true position often is, that of numbers who undergo such treatment the chance of any one individual suffering harm is only a small one. But the harm when it occurs may be disastrous. To state this is both right and necessary, whilst to use terms which suggest that mischievous results are comparatively common and widespread is to incur the chance of a rebuke from experience. It is advisable for the medical profession, as for the public, to look on both sides of the shield.

